# Diagnostic value of serum cystatin C for diabetic nephropathy: a meta-analysis

**DOI:** 10.1186/s12902-022-01052-0

**Published:** 2022-06-02

**Authors:** Xueling Liao, Yan Zhu, Chao Xue

**Affiliations:** 1 Department of Nephrology, the Second Affiliated Hospital of Guangxi Medical University, Nanning, 530007 Guangxi China; 2 Department of Nephrology, Affiliated Hospital of Guilin Medical University, Guilin, 541001 Guangxi China

**Keywords:** Cystatin C, Diabetic nephropathy, Diagnostic value, Meta-analysis

## Abstract

**Background:**

Although dozens of studies have investigated the relationship between the content of serum cystatin C (Cys-C) and diabetic nephropathy (DN), the results are still controversial. Hence, This study aims to explore the accuracy of serum Cys-C for diagnosing DN by meta-analysis.

**Methods:**

The studies about serum Cys-C diagnosing DN were searched from six online databases from inception to September 22, 2020. The data were processed by Stata 15.0 statistic software. The corresponding diagnostic effect sizes, such as sensitivity and specificity, were obtained. We drew a summary receiver operating characteristic (SROC) curve. We assess the risk of literature bias was following the QUADAS-2 guidelines.

**Results:**

Twenty-six published studies were identified. The results showed a pooled sensitivity of 0.86 (95% confidence interval (CI): 0.82–0.90), specificity of 0.89 (95%CI: 0.85–0.92), positive likelihood ratio of 7.59 (95%CI: 5.66–10.19), negative likelihood ratio of 0.16 (95%CI: 0.12–0.21), and diagnostic odds ratio of 48.03 (95%CI: 30.64–75.29). The area under the SROC curve was given a value of 0.94 (95%CI: 0.91–0.96).

**Conclusion:**

Serum cystatin C has an excellent diagnostic value with good sensitivity and specificity for diabetic nephropathy.

## Introduction

A total of 425 million people suffered from diabetes worldwide based on the International Diabetes Federation (IDF) (2017). The incidence will be increased to 629 million in 2045 if not controlled. There are about 842,993 deaths from diabetes in China, of which 33.8% patients are younger than 60 years (*IDF Diabetes Atlas. 8th Edition [EB / *OL]. [2019 - 08 - 14] http://www.idf.org/e-library/epidemiologyresearch/diabetes-atlas.html). Diabetic nephropathy (DN) is one of the most common serious complications of diabetes [1]. DN refers to kidney damage caused by chronic hyperglycemia, which becomes the leading cause of an end-stage renal disease (ESRD) in China instead of glomerulonephritis-related chronic kidney disease (CKD) [2]. Due to its insidious onset and slow development, the entire course of the disease could be irreversible at diagnosis, which led to disability and death eventually [3]. Therefore, the early diagnosis of DN is of significance for its treatment and prognosis [4]. With the extensive development of kidney biopsy, studies have found that diabetic patients with albuminuria or abnormal renal function do not necessarily have DN [5], which indicates the difficulty of early diagnosis of DN and the complicated disease development.

At present, two main clinical indicators including urine albumin and estimated glomerular filtration rate (eGFR) are used to diagnose DN. Since 2002, Kidney Dialysis Outcomes Quality Initiative (KDOQI) guidelines have recommended the 24-h urine albumin as an indicator for evaluating kidney damage in the course of diabetes [6]. However, albuminuria has some deficiencies as an important diagnostic indicator. Albuminuria is neither a unique marker of diabetic kidney damage nor a unique marker of kidney damage. Additionally, 24-h urine microalbumin as a method for early diagnosis of kidney disease changes generally in the early stage of glomerulopathy [7]. Urine microalbumin is likely affected by menstrual period, urine retention, blood pressure, exercise, urinary tract infection, and other factors that cannot fully meet clinical requirements [8]. Albuminuria can’t be detected in about 30% of diabetic patients who have developed renal failure [9]. GFR is mainly estimated by the serum creatinine concentration, which is likely affected by many other factors, such as muscle content, gender, age, diet, and medication. Apart from glomerular filtration, part of urine creatinine comes from the secretion of renal tubules. Therefore, the GFR estimated by the creatinine clearance level may be overestimated [10]. Therefore, the identification of non-invasive diagnostic markers with good sensitivity and specificity is the development direction of clinical nephrology. With the increase of patients with DN, it is necessary to explore non-invasive markers that reflect predictable and therapeutic effects.

Cystatin C (Cys-C) is a non-glycosylated low-molecular-weight (13 kDa) protein, whose concentration in serum is closely related to the GFR [11]. It stably exists in almost all nucleated cells in the human body with no tissue specificity, independent of gender, age, inflammatory state, and activity. The kidney is the only organ that clears Cys-C from the circulatory system, and the GFR mainly determines the concentration of serum Cys-C [12–14]. Prior reports [15, 16] have demonstrated that Cys-C can serve as an indicator for kidney function with close relation to GFR and good sensitivity regardless of mild, moderate, or severe renal dysfunction, suggesting its promise as a diagnostic marker.

Although there are many investigations on the association of serum Cys-C with patients with DN, major investigations are of discrepancy. To explore more objective evidence of the serum of Cys-C for diagnosing DN, we comprehensively searched the relevant studies and performed this meta-analysis.

## Methods

### Retrieval strategy of the literature

Two researchers retrieved relative studies about serum Cys-C in the diagnosis of DN independently from databases including Embase, Cochrane Library, Web of Science, PubMed, China National Knowledge Infrastructure (CNKI) and WanFang database from inception to September 22, 2020, with no limitations on language. The literature search formula was as follows: ("cystatin C" OR "Cys-C") AND ("diabetic nephropathy" OR "DN" OR "diabetic kidney disease" OR "DKD" OR "kidney" OR "renal function").

### Literature screening

#### Inclusion criteria:

(1) The data of serum Cys-C level could be collected; (2) The samples were enrolled from diabetic patients; (3) The enrolled diabetic patients were diagnosed with nephropathy; (4) The level of glomerular filtration rate (GFR), albumin-creatinine ratio (ACR), or albumin excretion rate (AER) in the patients with DN was provided.

#### Exclusion criteria:

(1) Case report, review, letter, conference abstract, or animal studies; (2) Insufficient data to extract to extract four-cell table data; (3) Duplicate data.

### Literature quality assessment

Two researchers evaluated the bias risk of the included literature according to the Quality Assessment of Diagnostic Accuracy Studies-2 (QUADAS-2) [17]. The scoring system contains 11 items, covering several aspects of the case selection, trials assessment, gold standard, case processes, etc. According to the answers to the landmark questions included in each part of "yes," "no," or "unclear," the risk of bias can be judged as "low," "high," or "moderate." Disagreements between the two authors were settled through discussion.

### Data retrieval

The information including the first author, year, region, type of diabetes, the method of Cys-C detection, number of participants, cut-off value, false negative, true negative, true positive, false positive, sensitivity (Sen), specificity (Spe), and diagnostic criteria for DN was extracted. Data retrieval was conducted independently by two researchers. A third author participated in the discussion in case of disagreement.

### Statistical analysis

The data analysis was processed by the Stata 15.0 statistical software [18]. The I^2^ index and *p*-value were used to assess the heterogeneity. I^2^ > 50% (*P* < 0.05) means significant heterogeneity[19]. We combined a typical "shoulder-arm" shape in the summary receiver operating characteristic (SROC) curve with the spearman correlation coefficient of the logarithm of 1-specificity with the logarithm of sensitivity to determine the threshold effect. Adopting the statistic model of bivariate mixed effects, we analyzed the following diagnostic effect sizes from positive likelihood ratio (+ LR), negative likelihood ratio (-LR), sensitivity, and specificity to diagnostic odds ratio (DOR), obtaining the corresponding forest plots [20]. The area under the curve (AUC) value was estimated [21]. The sources of heterogeneity were analyzed using Meta-regression, and the stability of the conclusion was assessed via sensitivity analysis. The assessment of the publication bias was performed by the asymmetry test of Deeks’ funnel plot. P-value < 0.05 is considered significant.

## Results

### Results and characteristics of the included articles

**Fig. 1:**
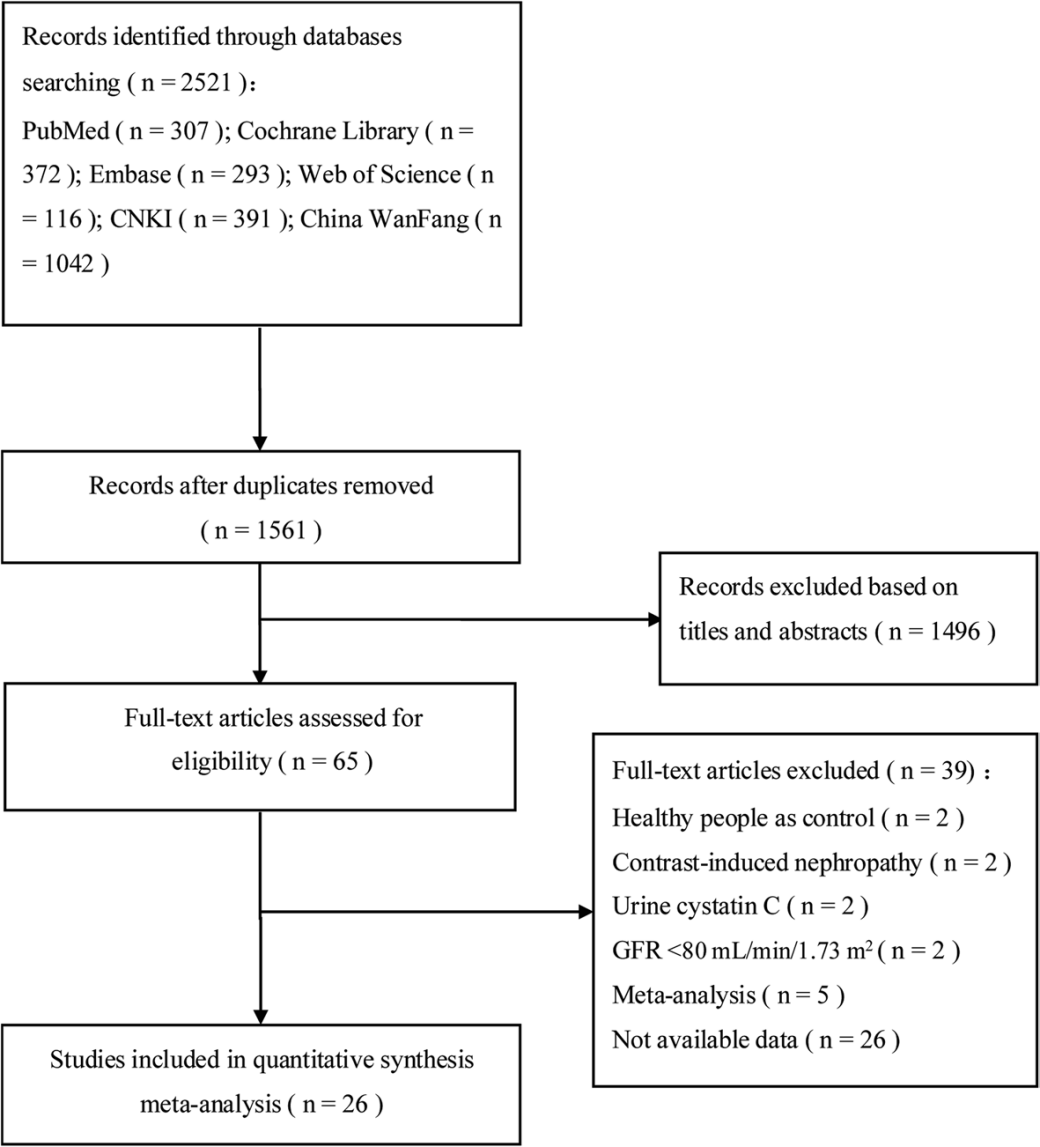
Flow diagram of study selection based on the inclusion and exclusion criteria. GFR: Glomerular Filtration Rate; CNKI: China National Knowledge Infrastructure

A total of 2521 published studies (PubMed 307, Cochrane Library 372, Embase 293, Web of Science 116, CNKI 391, and China WanFang 1042) were obtained after retrieval, among which 960 repeated ones. After reviewing the title and abstract, 1496 irrelevant articles were excluded, and after reviewing the whole text and complete data, 39 articles were excluded. Finally, 26 articles were included [22–47]. There were 3993 samples included in these studies, containing 1828 in the DN group, and 2165 controls. The detailed screening procedures were given in Fig. 1.

Among the 26 articles, 18 articles were published in English [22–34, 36, 42, 43, 45, 46], the other 8 articles published in Chinese [35, 37–41, 44, 47]. The enrolled patients were diagnosed with DN according to the GFR, ACR, and AER values. The main characteristics of the identified research were given in Table 1.

**Table 1:** Characteristics of the included studies

First author	Year	Region	Diabetic nephropathy type	Cys-C detection method	case (control)	Cut-off value	TP	FP	FN	TN	Sensitivity	Specificity	reference for GFR/ACR/AER
Mojiminiyi [22]	2000	Kuwait	T2DM	PENIA	50(27)	NR	20	3	30	24	0.4	0.89	ACR
Oddoze C [23]	2001	France	T1DM, T2DM	PENIA	10(39)	0.96 mg/L	9	4	1	35	0.9	0.9	GFR
Christensson AG [24]	2004	Sweden	T1DM, T2DM	PETIA	15(108)	NR	14	3	1	105	0.9333	0.9722	GFR
Bicik Z [25]	2005	Turkish	T2DM	PETIA	32(40)	1.4 mg/L	29	4	3	36	0.9	0.9	GFR
Beauvieux MC [26]	2007	France	T1DM, T2DM	PENIA	76(48)	0.96 mg/L	70	6	6	42	0.921	0.875	GFR
Macisaac [27]	2007	Australia	T1DM, T2DM	PENIA	54(197)	1.1 mg/L	53	20	1	177	0.981	0.898	GFR
Pucci [28]	2007	Italy	T1DM, T2DM	PENIA	218(70)	1.23 mg/L	168	2	50	68	0.77	0.97	GFR
Kimura [29]	2008	Japan	T2DM	Latex agglutination test	118(171)	0.85 mg/L	111	15	7	156	0.94	0.91	GFR
Rigalleau V [30]	2008	France	T1DM, T2DM	PETIA	76(48)	1.10 mg/L	70	9	6	39	0.921	0.8125	GFR
Iliadis F [31]	2011	Greece	T2DM	PETIA	145(303)	NR	118	61	27	242	0.812	0.798	GFR
Jeon [32]	2011	Korea	T2DM	Latex agglutination test	29(181)	1.06 mg/L	23	23	6	158	0.81	0.871	GFR
Bevc S [33]	2012	Slovenia	T2DM	PENIA	86(27)	NR	73	0	13	27	0.849	1	GFR
Chae HW [34]	2012	Korea	T1DM, T2DM	PENIA	20(93)	NR	17	31	3	62	0.873	0.662	GFR
Wang H [35]	2012	China	T2DM	NR	60(55)	1.55 mg/L	52	3	8	52	0.867	0.95	AER
Assal [36]	2013	Egypt	T2DM	PETIA	25(20)	2.45 mg/L	18	3	2	17	0.708	0.833	NR
Tan TT [37]	2015	China	T2DM	NR	47(111)	1.48 mg/L	38	11	9	100	0.809	0.904	ACR
Cao YY [38]	2015	China	T2DM	Turbidimetric inhibition immunoassay	60(43)	0.86 mg/L	56	3	4	40	0.9333	0.9277	ACR
Yang N [39]	2017	China	T1DM, T2DM	NR	103(85)	1.06 mg/L	88	29	15	56	0.8564	0.6531	ACR
Zhang HF [40]	2018	China	T2DM	Immune colloidal gold technique	265(165)	1.1 mg/L	176	9	89	156	0.665	0.945	ACR
Zhang RL [41]	2018	China	T2DM	NR	100(100)	NR	79	26	21	74	0.789	0.744	NR
Mohammed [42]	2019	Egypt	T2DM	PENIA	20(30)	1.6 mg/L	19	2	1	28	0.96	0.94	ACR
Xu WH [43]	2019	China	T2DM	NR	25(28)	1.12 mg/L	20	3	5	25	0.8	0.8929	AER
Wang HF [44]	2019	China	T2DM	PENIA	28(30)	1.75 mg/L	23	3	5	27	0.829	0.895	ACR
Salem [45]	2020	Egypt	T1DM	ELISA	31(29)	0.605 mg/L	27	5	4	24	0.883	0.833	ACR
Wang SY [46]	2020	China	T1DM, T2DM	Immunoturbidimetry assay	67(65)	2.35 mg/L	52	5	15	60	0.7835	0.9217	NR
Guang SF [47]	2020	China	T2DM	PENIA	68(52)	1.57 mg/L	63	17	5	35	0.926	0.673	AER

*TP* true positive, *FP* false positive, *FN* false negative, *TN* true negative, *NR* not reported, *T1DM* type 1 diabetic mellitus, *T2DM* type 2 diabetic mellitus, *Cys-C* cystatin C, *PENIA* particle enhanced nephelometry immunoassay, *PETIA* particle-enhanced turbidimetric immunoassay, *NR* not report, *GFR* Glomerular Filtration Rate, *ACR* Albumin/Creatinine Ratio, *AER* Albumin Excretion Rate

### QUADAS-2 scores

**Fig. 2:**
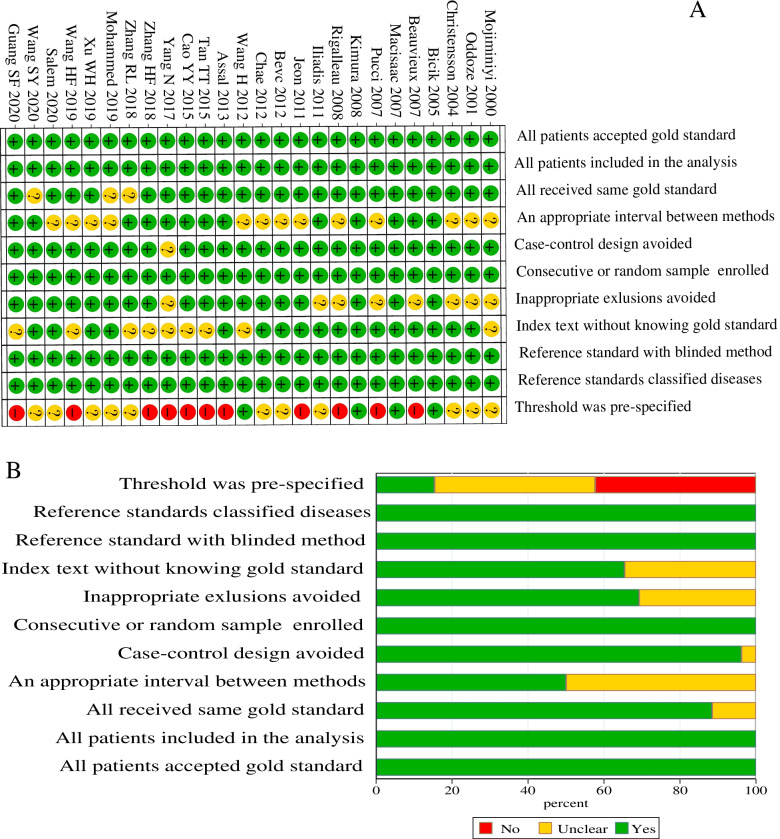
Quality assessment for the included studies following QUADAS-2. **A**. Risk of bias summary diagram. **B**. Risk of bias graph. QUADAS-2: Quality Assessment of Diagnostic Accuracy Studies-2

The results of bias risk assessment of the identified articles were provided (Fig. 2 A,B). Most of the included articles reached a medium-to-high quality level. All samples were selected continuously or randomly. The gold standards of all results were assessed blindly, and the gold standard can correctly distinguish the target disease state. Almost all studies avoided the case–control comparative study design. However, bias was found during experiment evaluation in terms of case processes and disease progression. For example, the duration of the research experiment was different. On the other hand, the threshold was not pre-specified.

### Meta-analysis results

**Fig. 3:**
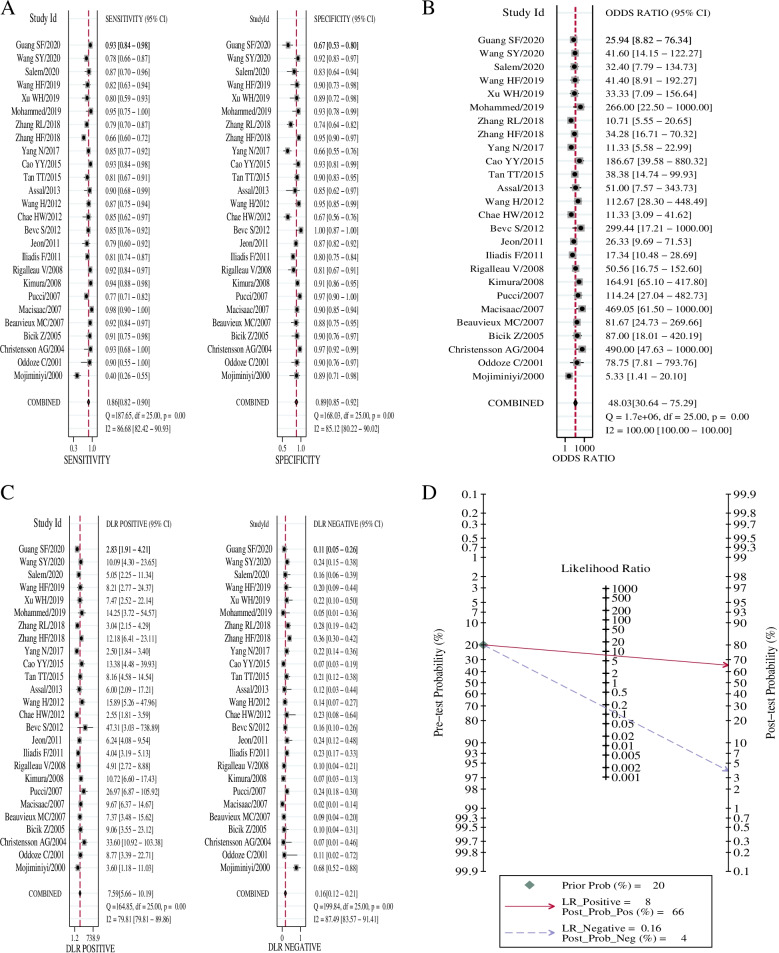
Diagnostic performance of serum cystatin C in diabetic nephropathy. **A**: Forest plots of sensitivity and specificity; **B**: Forest plots of + LR and -LR; **C**: Forest plot of DOR. **D**: Fagan’s Nomogram plot. + LR: Positive Likelihood Ratio; -LR: Negative Likelihood Ratio; DOR: diagnostic odds ratio

The heterogeneities were significant in the pooled analysis of sensitivity (*P* = 0.00, I^2^ = 86.68) (Fig. 3A), specificity (*P* = 0.00, I^2^ = 85.12%) (Fig. 3A), DOR (*P* = 0.00, I^2^ = 100%) (Fig. 3B), + LR (*P* = 0.00, I^2^ = 79.81%) (Fig. 3C), and -LR (*P* = 0.00, I^2^ = 87.49%) (Fig. 3C).

**Fig. 4:**
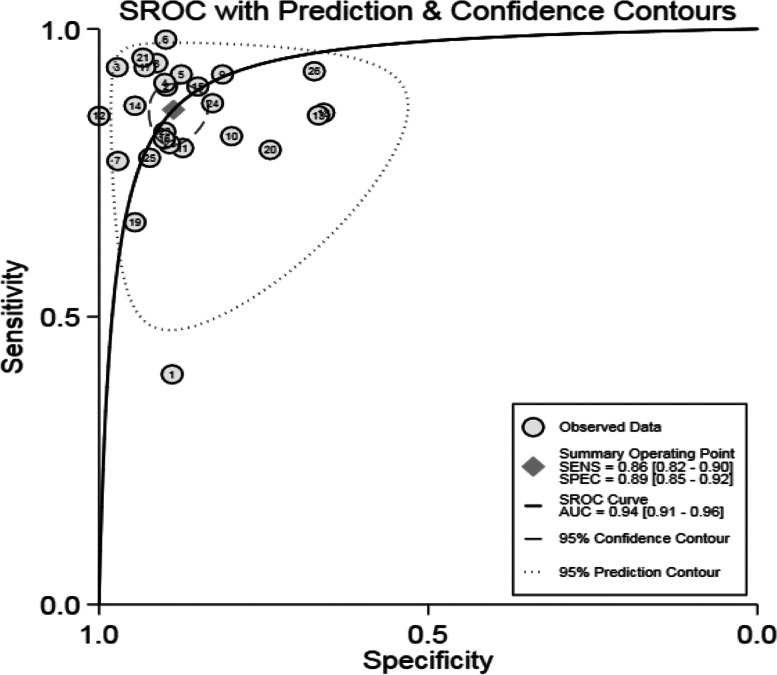
The SROC curve of serum cystatin C diagnosing diabetic nephropathy. SROC: summary receiver operating characteristic

A typical "shoulder-arm" shape of SROC curve did not display, with the Spearman correlation coefficient 0.061 (*P* = 0.776), indicating no obvious threshold effect. The bivariate mixed-effects model results showed the pooled Sen of 0.86 (95% confidence interval (CI): 0.82–0.90), Spe of 0.89 (95%CI: 0.85–0.92), + LR of 7.59 (95%CI: 5.66 10.19), -LR of 0.16 (95%CI: 0.12–0.21), and DOR of 48.03 (95%CI: 30.64–75.29). The Fagan’s Nomogram plot suggested that, when the probability ratio pre-test was given a value of 20%, the + LR probability post-test was 66%, the -LR post-test being 4% (Fig. 3D). The AUC was given a value of 94% (95% CI: 0.91–0.96) (Fig. 4). This indicates that serum Cys-C has excellent diagnostic accuracy for DN.

### Publication bias

**Fig. 5:**
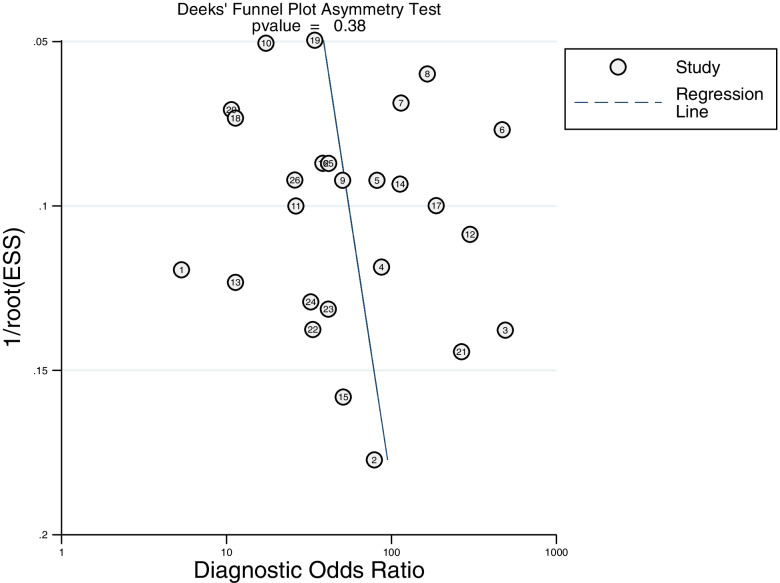
Deeks’ funnel plot asymmetry test for publication bias

No obvious publication bias existed in the asymmetry test of Deeks’ funnel plot (*P* = 0.38) (Fig. 5).

### Meta-regression and subgroup analyses

**Fig. 6:**
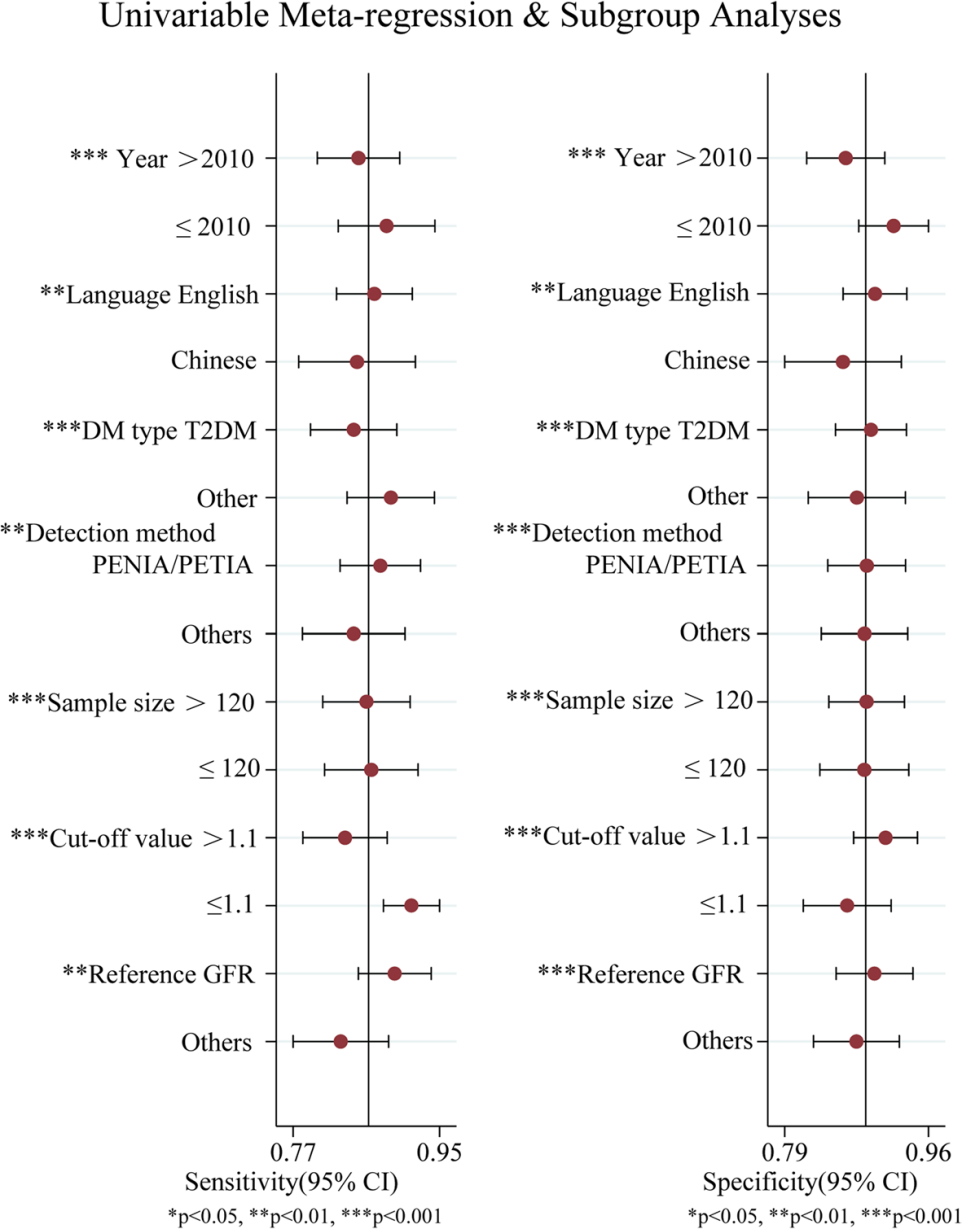
Meta-regression and subgroup analysis of serum cystatin C diagnosing diabetic nephropathy. PENIA: particle enhanced nephelometry immunoassay; PETIA: particle-enhanced turbidimetric immunoassay; T2DM: type 2 diabetic mellitus; GFR: Glomerular Filtration Rate; DM: diabetic mellitus

The article publication year, language, type of diabetes, Cys-C detection method, sample size, cut-off value, and diagnostic criteria were enrolled in the meta-regression and subgroup analyses (Fig. 6). The results showed that these factors could lead to a significance (*P* < 0.05), which might be the source of heterogeneity. The subgroups with the publication before 2010, the publication in English, the sample not limited to the type 2 diabetes subgroup, the detection method of PENIA or PETIA, the sample size ≤ 120 patients, the cut-off value ≤ 1.1 mg/L, and GFR as diagnostic criteria had higher sensitivity of statistical significance than the corresponding subgroups.

### Sensitivity analysis

**Fig. 7:**
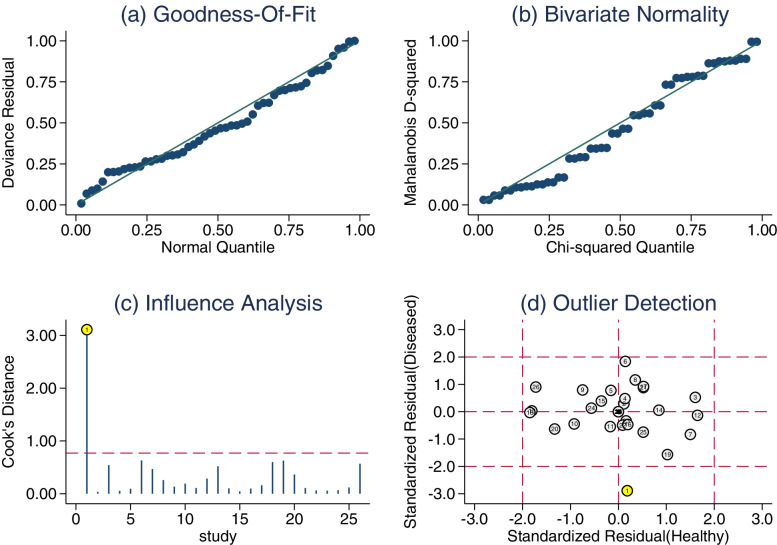
Sensitivity analysis of serum Cystatin C diagnosing diabetic nephropathy. **a**: Goodness of fit. **b**: Bivariate normality. **c**: Influence analysis. **d**: Outlier detection

Both goodness-of-fit and bivariate normality fit well (Fig. 7A, B). The impact analysis found a study [22] weight (Fig. 7C). The outlier detection showed that this study might be the sources of heterogeneity (Fig. 7D). After removing this abnormal article, the pooled sensitivity varied from 0.86 to 0.87; the specificity remained unchanged; the DOR increased from 48.03 to 51; the + LR increased from 7.59 to 7.6; the -LR decreased from 0.16 to 0.15. These data suggested that re-analysis was changed mildly compared with the combined results before exclusion. This indicates that the conclusion of this study are of robustness.

## Discussion

The pathogenesis of DN is quite complicated, involving genetic factors and metabolic disorders. Metabolic abnormalities caused by hyperglycemia, abnormal metabolism of vasoactive substances during the progression of diabetes, changes in kidney hemodynamics, albuminuria after kidney damage are the factors that cause glomerular basement membrane thickening, mesangial cell proliferation, and glomerular sclerosis [48], and finally leading to end-stage renal failure and death [49]. The clinical onset of DN is generally insidious, and the disease progresses slowly, which brings great difficulties in the early treatment of patients [50]. Kidney disease can be reversed after timely and effective symptomatic treatment [51]. However, when patients have symptoms of edema or obvious albuminuria, the optimal treatment time is missed out [52]. In 2014, the American Diabetes Association (ADA) and the National Kidney Foundation (NKF) reached a consensus. DN is defined as chronic kidney disease caused by diabetes, with symptoms mainly including GFR lower than 60 mL/min/1.73 m^2^ or the urinary ACR higher than 30 mg/g for more than three months [53, 54]. The tubular interstitial diseases share a closer association with kidney damage caused by DN than glomerulus, and tubular damage appears in the early stage of DN, before the glomerular disease [55]. The use of eGFR alone for diagnosis of diabetes combined with CKD is only suitable for patients with advanced-stage (≥ stage III), early diagnosis of CKD requires the detection of other markers for early kidney damage [56]. The early detection of DN mainly focuses on the urine protein excretion rate. However, 20%-30% of patients with type 2 diabetes have already suffered kidney damage even when their urine protein excretion is normal [57]. With the continuous improvement of the Cys-C standardization system, CKD-EPI was published in 2012 based on the Cys-C or combined Cys-C and Cr eGFR formula. Many studies have shown that it evaluates glomerular filtration function more precisely [58]. Serum Cys-C, as a sensitive indicator of early kidney damage, can accurately reflect GFR [59].

The pooled Sen and Spe in the meta-analysis were 0.86 and 0.89, respectively, suggesting that serum Cys-C has good sensitivity and specificity for diagnosing DN. The + LR and -LR were 7.59 and 0.16, respectively, indicating that patients with DN were 7.59 times more likely to be correctly diagnosed as positive than misdiagnosed as positive, while the likelihood of patients being wrongly judged negative was 16% of the likelihood of being correctly judged negative. The + LR > 10, and the -LR < 0.1 indicate convincing diagnostic performance [60], suggesting that serum Cys-C is of limitation in the diagnosis of DN. An increasing DOR value (0 to infinity) means better diagnostic potential [61]. The DOR value in this study was 48.03, suggesting that serum Cys-C is a biomarker for diagnosing DN. AUC more than 0.9 means excellent diagnostic capabilities [62, 63]. The AUC in this meta-analysis was 0.94, suggesting that serum Cys-C has a promising diagnostic accuracy for DN, which was consistent with the findings of the reviews in 2016 [64, 65]. According to a more strict standard, new research after 2016 were enrolled in our study [64, 65].

Meta-regression and subgroup analyses suggested that publication year, publication language, type of diabetes, Cys-C detection method, sample size, cut-off value, and diagnostic criteria might be the sources of heterogeneity. Higher diagnostic value was found in the groups with publication year ≤ 2010 group, publication in English, samples not limited to type 2 diabetes, PENIA, or PETIA for detection of serum Cys-C, sample size ≤ 120, cut-off value ≤ 1.1 mg/L, and GFR as diagnostic criteria than that of the corresponding group. The sensitivity analyses and publication bias suggested that the findings were stable and credible in this meta-analysis.

There were also some limitations in our study. First of all, the included studies included comparative studies of Cys-C and other molecules in the diagnosis of DN and combined diagnostic value studies. The inclusion criteria used between the trials, the staging of DN, the presence of other combined diseases, sample size, detection methods, and the choice of the gold standard are different, leading to the heterogeneity of results. Secondly, some of the included studies did not describe in detail information, such as the trial randomization, blinded design, and quality control, which might affect the quality of this study. Finally, the included articles were from multiple countries, and the incidence and medical level were different among different countries and regions, which could affect the accuracy of the diagnosis and thus affect the results of this study. Therefore, the investigation of the correlation between Cys-C in the diagnosis of DN requires a large sample, random, blinded research design, using a unified gold standard and disease staging, so that the authenticity and reliability of the results are more clinically meaningful.

## Conclusion

In summary, this meta-analysis indicates that serum cystatin C has an excellent diagnostic value with good sensitivity and specificity for patients with DN. This study reveals an association of serum Cys-C with patients with DN. Serum Cys-C is conducive to the diagnosis of this disease. Considering the limitations of this meta-analysis, the conclusions of this research are yet to be confirmed using high-quality clinical trials in the future.

## Data Availability

The datasets using in the current meta-analysis are available on reasonable request from the corresponding author.

## Abbreviations

Sen: Sensitivity
Spe: Specificity
DOR: Diagnostic Odds Ratio
+ LR: Positive Likelihood Ratio
-LR: Negative Likelihood Ratio
AUC: Area Under the Curve
ROC: Receiver Operating Characteristic Curve
SROC: Summary Receiver Operating Characteristic Curve
PETIA: Particle-Enhanced Turbidimetric Assay
PENIA: Particle Enhanced Nephelometry Assay
ADA: American Diabetes Association
NKF: National Kidney Foundation
Cys-C: Cystatin C
DN: Diabetic Nephropathy
IDF: International Diabetes Federation
ESRD: End-Stage Renal Disease
CKD: Chronic Kidney Disease
eGFR: Estimated Glomerular Filtration Rate
KDOQI: Kidney Dialysis Outcomes Quality Initiative
ACR: Albumin-Creatinine Ratio
AER: Albumin Excretion Rate
QUADAS-2: Quality Assessment of Diagnostic Accuracy Studies-2
CNKI: China National Knowledge Infrastructure
TP: True Positive
FP: False Positive
FN: False Negative
TN: True Negative
